# Predicting regional carbon price in China based on multi-factor HKELM by combining secondary decomposition and ensemble learning

**DOI:** 10.1371/journal.pone.0285311

**Published:** 2023-12-12

**Authors:** Beibei Hu, Yunhe Cheng

**Affiliations:** School of Economics and Management, Anhui University of Science and Technology, Huainan, China; Univerzitet Singidunum, SERBIA

## Abstract

Accurately predicting carbon price is crucial for risk avoidance in the carbon financial market. In light of the complex characteristics of the regional carbon price in China, this paper proposes a model to forecast carbon price based on the multi-factor hybrid kernel-based extreme learning machine (HKELM) by combining secondary decomposition and ensemble learning. Variational mode decomposition (VMD) is first used to decompose the carbon price into several modes, and range entropy is then used to reconstruct these modes. The multi-factor HKELM optimized by the sparrow search algorithm is used to forecast the reconstructed subsequences, where the main external factors innovatively selected by maximum information coefficient and historical time-series data on carbon prices are both considered as input variables to the forecasting model. Following this, the improved complete ensemble-based empirical mode decomposition with adaptive noise and range entropy are respectively used to decompose and reconstruct the residual term generated by VMD. Finally, the nonlinear ensemble learning method is introduced to determine the predictions of residual term and final carbon price. In the empirical analysis of Guangzhou market, the root mean square error(RMSE), mean absolute error (MAE) and mean absolute percentage error (MAPE) of the model are 0.1716, 0.1218 and 0.0026, respectively. The proposed model outperforms other comparative models in predicting accuracy. The work here extends the research on forecasting theory and methods of predicting the carbon price.

## 1 Introduction

The warming climate threatens human health and sustainable development. It is mainly due to the increasing of carbon dioxide (CO_2_) concentration in the atmosphere. According to the report released by the national oceanic and atmospheric administration of the United States in 2022, the global average concentration of CO_2_ in the atmosphere was 421 ppm, nearly 50% higher than the concentration of 280 ppm before industrialization. Based on the statistical review of world energy released in 2022, the CO_2_ emissions of China accounted for 30.89% of the global total, making it the largest emitter of the CO_2_ [1]. Therefore, it is urgent for China to reduce CO_2_ emissions. In order to promote carbon emissions reduction, China has formed the "7+2" development pattern of regional carbon markets. Specifically, it has established seven pilot carbon markets and two non-pilot carbon markets [2]. Moreover, regional carbon markets are conducive to promoting the growth of low-carbon industries as well as energy transformation. China’s national carbon market commenced online trading in July 2021. The carbon market is important for China to realize its aims at achieving the carbon peak and carbon neutralization. It is noteworthy that the national market is immature in market operation and system design, which requires regional carbon emissions trading markets to accumulate operational experience [3].

Carbon market price is one of the core indicators of the carbon market. According to the Kyoto Protocol, the carbon emission quotas traded in financial markets have commodity-related as well as financial attributes [4]. Its price can indicate the cost of carbon emission abatement for the economy [5]. It can compel enterprises to optimize their resource allocation to achieve their emissions reduction targets at the lowest cost [6]. However, in the complex global economic environment, China’s carbon market price fluctuates sharply and transaction risks increase. The drastic fluctuation of the carbon market price is too high or too low, which is not conducive to the long-term stable operation of the carbon market. Reasonable carbon emission quota pricing is an effective means of reducing greenhouse gas emissions [7], which will provide an effective price incentive signal for emission reduction enterprises. Consequently, accurate predicting of carbon price can provide valuable information for market participants to manage associated risks resulted from changes in price and for policy-makers to formulate relevant policies.

Carbon prices exhibit nonlinear and nonstationary characteristics due to various factors [8]. It makes them challenging to predict. The aim of this research is to develop a combined framework for forecasting the regional carbon prices. The main contributions of this article can be illustrated as follows: (1) The multi-factor HKELM model is introduced to forecast carbon price. External factors influencing the carbon price and historical data on it are taken as input of the HKELM model to make the expression capability of the forecasting model of China’s regional carbon price closer to reality. (2) Ensemble learning based on the SSA-HKELM is introduced to integrate the results of prediction of each subsequence of carbon price generated by secondary decomposition to obtain the final predictions. This can help distinguish between the impacts of different subsequences on the overall results of prediction. (3) The maximum information coefficient (MIC) is innovatively used to select the main factors influencing China’s regional carbon price to realize the purpose of dimension reduction, which can in turn capture the nonlinear and linear relationships between external factors and carbon price.

The rest of the study is as follows. Section 2 provides the literature review. Section 3 contains the theoretical methods and the framework of the carbon price forecasting model. Section 4 introduces the empirical analysis of the predicting of carbon price in China. Section 5 contains the main conclusions and the prospects.

## 2 Literature review

Current research on predicting the price of carbon in regional markets in China can be mainly divided into two dimensions: the prediction of carbon prices based on historical time-series data, and prediction considering factors influencing this price.

### 2.1 Research on carbon price prediction based on historical time series of carbon price

Existing forecasting methods of China’s carbon price according to historical time series contain two types: single models and combined models. Single models, containing the generalized autoregressive conditional heteroscedasticity [9], along with RBF neural network [10], have been used to predict pilot carbon prices in China. However, single model homogenizes information on the different characteristics of carbon price, which struggles to fully depict the price.

Regional carbon price in China is characterized by nonlinearity, nonstationarity, asymmetry as well as a wide range [11]. The combination of models with signal decomposition technology, which can mine the internal laws and essential characteristics of the data on carbon price at different frequencies, has become the mainstream method for forecasting carbon price. As a classical signal decomposition technology, empirical mode decomposition (EMD) has been widely used to smooth the non-stationary carbon price data. For example, Li and Lu (2015) [12] applied EMD to preprocess the carbon price and then used the GARCH model to forecast it. However, EMD has the problem of mode aliasing. Some more advanced signal decomposition technologies came into being. Sun and Xu (2021) [13] applied ensemble-based empirical mode decomposition (EEMD) and LSSVM to forecast carbon price, and showed that signal decomposition can weaken the complexity of carbon price and LSSVM can yield more accurate forecasts than BP. However, EEMD still has some remnant noise. Wang et al. (2021) [14] used the complete ensemble-based EMD with adaptive noise (CEEMDAN) to decompose carbon price and used the long short-term memory (LSTM) to forecast it, and the results verified that the CEEMDAN outperforms EEMD. However, CEEMDAN still has the drawback of slight remnant noise [15]. The combined model of the improved complete ensemble-based empirical mode decomposition with adaptive noise (ICEEMDAN) and extreme learning machine (ELM) was employed to predict the carbon price, proving that ICEEMDAN can get more regular mode components than CEEMDAN [16]. VMD can decompose carbon price more regularly and avoid modal aliasing. Niu et al. (2022) [17] predicted carbon prices via the combined model of VMD, sample entropy (SE), and outlier robust extreme learning machine, demonstrating that the decomposition effect of VMD is superior to that of EEMD and EMD. Wang et al. (2022) [18] forecasted carbon price via the combined model of VMD, multiscale entropy and ELM optimized by SSA, and proved that VMD can extract the hidden nonlinear characteristic in carbon price and SSA-ELM has stronger predictive accuracy.

However, a single decomposition-based strategy cannot completely deal with random and irregular time series, and yields large errors in the prediction of partially decomposed series [19]. The secondary decomposition strategy can better reduce the complexity of the data, and has been widely used for carbon price decomposition. Zhou et al.(2022) [20] predicted carbon prices via the combined model of the CEEMDAN, VMD, SE, and LSTM, proving the superiority of the secondary decomposition for carbon price forecasting and that VMD has effective decomposition effect on the most complex subseries obtained by CEEMDAN. Li et al.(2022) [21] predicted carbon prices through the combination of VMD, CEEMDAN, and PSO-ELM, and proved that the secondary decomposition strategy based on VMD-CEEMDAN can improve predictions of carbon price compared with single decomposition. Cheng and Hu (2022) [22] designed the combination of VMD, ICEEMDAN, range entropy (RE), and HKELM optimized by the SSA to predict carbon prices, proving that the secondary decomposition strategy of VMD-ICEEMDAN outperformed the VMD, and forecasts of carbon price by the HKELM are superior to those of the kernel extreme learning machine (KELM).

### 2.2 Research on carbon price prediction by considering multiple influential factors

The above research ignored the role of external factors on carbon price. External factors, including economic activities, energy price, and environmental factors, are significant sources of the uncertainty of the carbon price and can provide useful information related to carbon price forecasting [23–25]. Therefore, fluctuations in regional carbon price in China depend not only on their historical time series, but also on lots of external factors. Guo et al.(2022) [26] found that energy price can be utilized to predict regional carbon price. Based on the structural VAR model, Zeng et al [27] stated that the carbon price was correlated with its own historical price, domestic energy prices and economic factors. Sun and Zhang(2022) [28] designed a combination of local characteristic-scale decomposition, Pearson’s correlation coefficient, and LSSVM model by considering economic factors, energy-related factors, and variables of historical autocorrelation to predict carbon price, proving that the combined model has high predictive accuracy. However, the Pearson correlation coefficient cannot explain the nonlinear relationship between carbon price and external factors. Liu and Xu (2021) [29] found that the real economy, natural gas price and coal price exert non-linear impacts on China’s carbon price. Zhou et al. (2021) [30] applied VMD to process the high-frequency carbon price generated by EMD, and used the max-relevance min-redundancy (mRMR) algorithm to identify external factors affecting carbon price. They simultaneously considered such external factors as the price of coal, natural gas, CER, and the historical data on each subseries as input variables, and used a KELM optimized by the SSA to predict the subseries of carbon price. The conclusion verified that the forecasting performance of carbon price can be improved through the process of EMD-VMD and the consideration of influential factors. Hao and Tian (2020) [31] decomposed the price of carbon by ICEEMDAN, used mRMR to analyze the influence of external factors on it, and forecasted the carbon price by the KELM model. The results showed that energy-related factors, economic factors, international carbon price, and environmental factors, exert major impacts on improving the predictive performance of carbon price. However, the above research has ignored the impact of climate change on the carbon price. Existing studies have found that the fluctuations in the carbon price are quite sensitive to the air quality index (AQI), temperature [32], and that the prediction results of carbon price can be improved by incorporating climate-related variables [33].

In summary, the above literature has made considerable progress and provides strong theoretical support for the work here. But the following shortcomings persist: (1) Current studies have used the HKELM to forecast carbon price based only on historical time-series data, and have ignored the impact of external factors. (2) In research on prediction of carbon price based on secondary decomposition, the final prediction of carbon price, arrived at after having obtained the results of prediction of each subsequence of carbon price, is obtained by linear superposition without considering the influence of different subsequences on the overall results of prediction. This may affect the prediction accuracy. (3) Prevalent methods for selecting the variables of factors influencing carbon price have certain defects. Pearson’s correlation coefficient cannot identify the nonlinear relationship between carbon price and external influencing factors, and mRMR has such problems as incompatibility between the measures of correlation and redundancy [34].

To address the above research deficiencies, this paper constructs a hybrid model based on multi-factor HKELM by combining secondary decomposition and ensemble learning for forecasting regional carbon prices in China. Firstly, VMD and RE are used to preprocess the carbon price to reduce sequence complexity. Secondly, the multi-factor HKELM optimized by the SSA is introduced to forecast the subsequences, where the main external factors affecting the price of carbon are identified by the MIC method. Then, this paper combines ICEEMDAN, RE, and SSA-HKELM to forecast the residual term generated by VMD. Finally, the nonlinear ensemble learning method is introduced to determine the prediction of the final carbon price.

## 3 Methodologies

This section briefly introduces the technology of data decomposition and reconstruction, the feature selection, the SSA-HKELM model, and a framework of the proposed model.

### 3.1 Variational mode decomposition

Owing to the nonlinear and nonstationary fluctuations in carbon price, the extraction of data rules is particularly important. VMD [35] is a technique of signal processing. Compared with EMD, VMD has a more rigorous mathematical theoretical framework [36], and can overcome mode aliasing as well as signal noise [37]. Therefore, VMD is adopted to decompose the carbon price to reduce the difficulty of predicting it. Based on VMD, more regular and predictable intrinsic mode functions, denoted by VMF components, can be extracted. In terms of the VMD method, the residual term can be obtained by subtracting the sum of VMFs from the raw carbon price.

For the raw carbon price *y*, the VMD method can decompose it into several VMF components containing main information of the carbon price. The process of VMD is realized by solving the problem:

$$\begin{array}{l} \underset{\left\{y_{k}\},\{w_{k}\right\}}{\min}\{\sum_{k=1}^{K}{\left\|\partial_{t}[(\delta (t)+\frac{j}{\pi t})*y_{k}(t)]e^{-jw_{k}t}\right\|}_{2}^{2}\}\\ s.t.\;\sum_{k=1}^{K}y_{k}=y \end{array}$$ (1)

Where *y*_*k*_ is the k-th VMF, *w*_*k*_ represents its central frequency, *j* denotes $\sqrt{-1}$, *K* is the number of VMFs, *δ*(t) is the unit impulse function, ∂_*t*_ is the partial derivative of t, * is the convolution operation symbol, the exponential term $e^{-jw_{k}t}$ is added to modulate each single-sided spectrum to the corresponding fundamental frequency band.

Through introducing the Lagrange multiplier λ to turn the above problem into the following problem:

$$\begin{array}{c} L(y_{k},w_{k},\lambda )=\alpha \sum_{k=1}^{K}{\left\|\partial_{t}[(\delta (t)+\frac{j}{\pi t})*y_{k}(t)]e^{-jw_{k}t}\right\|}_{2}^{2}\\ +{\left\|y_{t}-\sum_{k=1}^{K}y_{k}(t)\right\|}_{2}^{2}+<\lambda (t),y(t)-\sum_{k=1}^{K}y_{k}(t)> \end{array}$$ (2)

Where α represents the data-fidelity constraint. The alternative direction method of multipliers is applied to solve the above equation. The following formulas are used to update the mode, its central frequency and λ:

$${\hat{y}}_{k}^{n+1}(w)=\frac{\hat{y}(w)-\sum_{i\neq k}^{n}{\hat{y}}_{i}^{n}(w)+\frac{{\hat{\lambda }}^{n}(w)}{2}}{1+2\alpha {\left(w-w_{k}^{n}\right)}^{2}}$$ (3)

$$w_{k}^{n+1}=\frac{{\int_{0}^{\infty }w{|\hat{y}}_{k}^{n}(w)|}^{2}dw}{{\int_{0}^{\infty }{|\hat{y}}_{k}^{n}(w)|}^{2}dw}$$ (4)

$${\hat{\lambda }}^{n+1}(w)={\hat{\lambda }}^{n}(w)+\tau (\hat{y}(w)-\sum_{k=1}^{K}{\hat{y}}_{k}^{n+1}(w))$$ (5)

where $\hat{y}(w)$, ${\hat{y}}_{k}(w)$, $\hat{\lambda }(w)$ and ${\hat{y}}_{k}^{n+1}(w)$ are the Fourier transforms of *y*(*w*), *y*_*k*_(*w*), *λ*(*w*), and $y_{k}^{n+1}(w)$, respectively. *n* is the number of iterations, *τ* is tolerance to noise that satisfies the requirements of fidelity of the decomposition of the carbon price.

The specific steps of the VMD are as follows:

Step1: Define the initial mode $y_{k}^{1}$, center pulsation $w_{k}^{1}$, and *λ*^1^.

Step2:Update the parameters *y*_*k*_ and *w*_*k*_ with Eqs (3) and (4).

Step3: Update the value of *λ* with Eq (5).

Step4: Given a convergence error *ε*. If the condition for stopping the iterations, $\sum_{k=1}^{K}\|{\hat{y}}_{k}^{n+1}-{{\hat{y}}_{k}^{n}\|}_{2}^{2}/{\|{\hat{y}}_{k}^{n}\|}_{2}^{2}<\varepsilon$, is satisfied, the process of VMD is over, otherwise, return to Step 2.

### 3.2 ICEEMDAN

The residual term of carbon price generated by the VMD fluctuates violently, and lacks regularity. We use decomposition technology to reduce the difficulty of predicting the residual term. The ICEEMDAN method is an improvement over CEEMDAN. It can eliminate residual noise and minimizes the overlays in the modes [38]. Therefore, ICEEMDAN is employed to preprocess the residual term into several intrinsic mode function (IMF).

**Fig 1:**
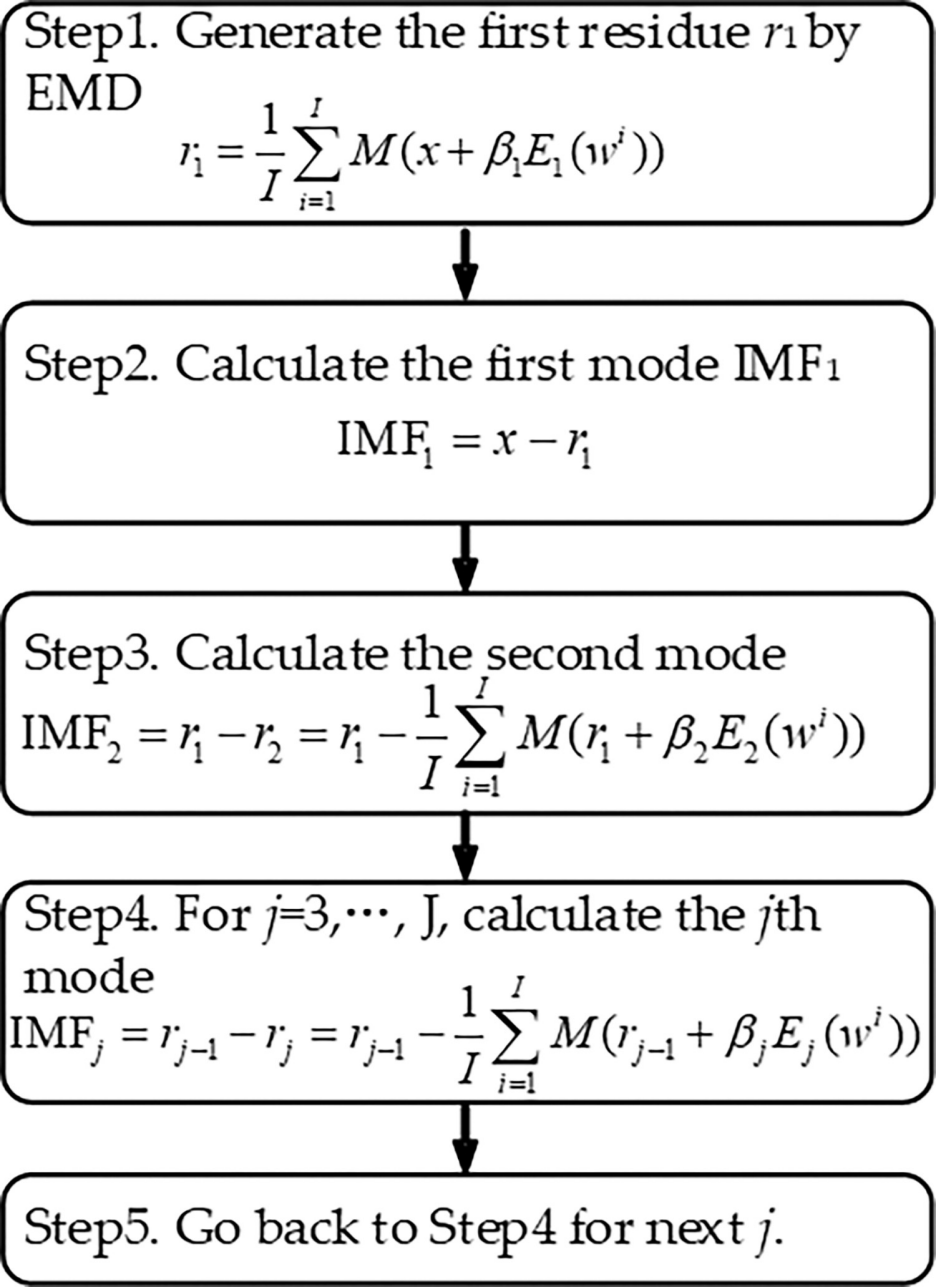
Implementation steps of ICEEMDAN.

Let *x* represent the original data of residual term. *j* = 1,2,..,*J*. Let *E*_*j*_() be the *j*-th IMF generated by EMD. Let *M*() be the local mean generated by the upper and lower envelopes of the signal. Let *w*^*i*^ represents white noise, and *β*_*j*_ controls the level of white noise. The specific procedures of the ICEEMDAN are shown in Fig 1.

### 3.3 Range entropy

Many relatively stable VMF and IMF components are obtained after secondary decomposition processing. However, if all the modes are forecasted respectively, forecasting error can easily accumulate. Thus, it is significant to reconstruct the modes to let the information of carbon price more aggregated, which reduces the accumulation of forecasting error. The complexity of the time series is commonly used as the basis for reconstruction, which can avoid the subjectivity of reconstruction process. The RE is developed based on the SE algorithm and applied for calculating complexity of time series. The robustness of the RE to changes in nonstationary signals is superior to the SE. Therefore, the reconstruction of these VMF and IMF components is carried out according to the RE. Specifically, the complexity of the modes is calculated by the RE. The more complex the time series of carbon price is, the larger is the range entropy. The simpler the fluctuation of carbon price is, the lower is the range entropy. In view of the strong regularity of simple time series, the sequences with lower results of RE can be combined into a new subseries to reduce the cumulative error of system prediction. The steps of the RE algorithm can refer to Omidvarnia et al.(2018) [39]. In general, the value of RE depends on the embedding dimension *m* and the tolerance *r*, where *m* and *r* are set to 2 and 0.5, respectively.

### 3.4 Feature selection of input variables

Fluctuations in China’s regional carbon prices depend on lots of factors. For instance, the price of energy, including coal, oil, and natural gas [40], macroeconomic conditions [41], the international price of carbon, weather conditions [42], carbon market policies [43], and historical data on carbon price. However, considering all factors to forecast the carbon price will cause redundancy of the model. The contents of the input for the model to predict carbon price play a decisive role in this regard. It is important to select factors that are highly correlated as the input variables to the prediction model. We select input variables based on the PACF and MIC.

#### 3.4.1 Partial autocorrelation function

If the lag in each subsequence of carbon price is unreasonably set, predictions of the carbon price are unfavorably influenced. The PACF can identify the degree of lag in the time series. Thus, it is applied to determine historical lag data closely related to each subsequence of carbon price.

#### 3.4.2 Maximum information coefficient

MIC, developed by Reshef [44], is an improved application of mutual information algorithm. MIC can measure correlation between two variables. Compared with the Pearson correlation coefficient and the generalized elastic network (GEN), the MIC can measure the nonlinear relationships between variables [45]. Moreover, it has high robustness and low computational complexity. The grid division is employed to calculate the MIC. The value of MIC is in the interval [0,1]. The larger the correlation between variables is, the higher is the MIC value. Therefore, the MIC is adopted to select effective external influencing factors as a part of input variables.

### 3.5 SSA-HKELM

This section introduces the forecasting model of carbon price and its key parameters optimization theory.

#### 3.5.1 Hybrid kernel-based extreme learning machine

ELM is a novel feedforward neural network. By comparison with traditional neural networks, ELM models have less parameter setting, faster learning rate, stronger generalization ability, simplicity, and ease of use [46,47]. However, the ELM model has a few shortcomings: (1) The input weights and hidden layer thresholds are created randomly; (2) The number of hidden layer nodes needs to be determined subjectively. These shortcomings will somewhat weaken the stability of the ELM. Huang et al [48] first developed KELM, which is an improvement based on ELM. In KELM, the kernel mapping replaces the random mapping. The generalization ability and stability of the KELM model is superior to ELM [49]. However, different kernel functions have significantly different forecasting performance. Any base kernel may not be suitable for a variety of applications. Usually, the KELM with a single kernel function has limited representation capability and struggles to capture the complicated characteristics in carbon price. Compared with KELM, the HKELM has better generalization performance and learning ability, and can enhance forecasting performance [50,51]. Therefore, the HKELM is used to forecast carbon price.

For the training dataset (*x*_*i*_,*t*_*i*_), the input included in the forecasting model is *x*_*i*_, *t*_*i*_ is its output. The standard KELM regression model can be displayed as:

$$f(x)=[\begin{array}{c} K(x,x_{1})\\ \vdots \\ K(x,x_{N}) \end{array}]{\left(I/C+\Omega_{KELM}\right)}^{-1}T$$ (6)

In Eq(6), **Ω**_*KELM*_ is a kernel matrix, *I* is a unit diagonal matrix, *C* represents a regularization coefficient, and **T** is the target output matrix.

The popular kernel functions used in the KELM model are RBF kernel and Poly-kernel, corresponding formulas are as follows:

$$K_{rbf}({x,x}_{i})=\exp(-\frac{∥{x-x}_{i}{∥}^{2}}{a})$$ (7)

$$K_{poly}({x,x}_{i})={\left(x\cdot x_{i}+b\right)}^{d}$$ (8)

In the above kernel functions, each kernel function has different computing capabilities and scope of application. For example, the RBF kernel has better learning capability and poor generalization capability, while the poly-kernel has strong generalization capability and poor learning capability [52]. Therefore, it is important for the KELM to determine a suitable kernel function. The hybrid kernel is formed by combining the poly-kernel and the RBF kernel, which can combine the advantages of both and get more accurate forecasting results. The hybrid kernel function is defined as follows:

$$K_{hybrid}({x,x}_{i})=L\times K_{rbf}({x,x}_{i})+(1-L)\times K_{poly}({x,x}_{i})$$ (9)

In Eq (9), *L* is the weight. Carbon price subsequence generated by decomposition and reconstruction contains various complex characteristics, and some subsequences show violent nonlinear random fluctuations. KELM with single kernel struggles to comprehensively describe these characteristics, and the artificial selection of single kernel function lacks objectivity. HKELM, which contains weighted kernel functions, can enhance the objectivity of kernel function selection. Therefore, this paper uses the HKELM model as prediction and ensemble learning tools. Specifically, the kernel function used in the HKELM model is the Eq (9). However, the key parameters included in the HKELM model, namely, *L*, *a*, *b*, *d*, and *C*, will deeply affect the forecasting performance of carbon price. It is noteworthy that inefficient optimization will lead to an imperfect HKELM model and poor forecasting capability. Therefore, it is necessary to effectively optimize these key parameters.

#### 3.5.2 Sparrow search algorithm

Metaheuristics can enhance the output over time to minimize errors [53]. When utilizing metaheuristics in prediction models, parameter tuning can produce better results [54]. Swarm intelligence provides an extremely potent group of metaheuristic optimization methods [55], often inspired by groups observed in nature such as SSA. As an optimization algorithm, SSA is proposed by Xue and Shen(2020) [56]. Compared with PSO, it has faster convergence, stronger optimization ability and stronger robustness [57]. Therefore, the above parameters of the HKELM are selected by the SSA.

In SSA, the results of optimization are obtained through simulating the process of sparrows foraging and anti-predatory behavior. According to the basic idea of SSA, the sparrow population is divided into three roles: discoverer, joiner and vigilant.

The discoverers actively look for food sources. In general, the discoverers account for 10% to 20% of the total. The formula for position iteration of the discoverers is:

$$x_{id}^{t+1}=\{\begin{array}{l} x_{id}^{t}*\exp(\frac{-i}{\alpha *T}),R_{2}<ST\\ x_{id}^{t}+Q*L,R_{2}\geq ST \end{array}$$ (10)

Where $x_{id}^{t}$ is the value of the *d* dimension of the *i* sparrow at the *t* iteration. *T* is the maximum iterations, *i* = 1,2,…,N, N is the number of sparrows, *α* and *Q* represent random numbers, *t* is the current times of iterations, *L* is a matrix whose all elements are 1, with a size of 1 × *d*, *ST* ∈ [0.5, 1] represents a safe value, and *R*_2_ represents a warning value between [0, 1]. When *R*_2_<*ST*, it indicates that the search environment is safe, there are no predators, and the discoverers will broaden the search area to obtain better fitness. When *R*_2_≥*ST*, predators are found around the foraging location, the population immediately adjusted the search strategy, and all sparrows should move to other safe place quickly.

The joiners follow the discoverer for food. The position update formula of the joiners is:

$$x_{id}^{t+1}=\{\begin{array}{l} Q*\exp(\frac{{xw}_{d}^{t}-x_{id}^{t}}{i^{2}}),i>\frac{n}{2}\\ {xb}_{d}^{t+1}+\frac{1}{D}\sum_{d=1}^{D}\left(rand\{-1,1\right\}*|x_{id}^{t}-{xb}_{d}^{t+1}|),i\leq \frac{n}{2} \end{array}$$ (11)

where ${xb}_{d}^{t+1}$ is the best position, and ${xw}_{d}^{t}$ represents the worst position.

Sparrows for early warning and reconnaissance usually occupy 10% to 20% of the entire population. These sparrows are called vigilantes. The position is updated as below:

$$x_{id}^{t+1}=\{\begin{array}{l} {xb}_{d}^{t}+\beta (x_{id}^{t}-{xb}_{d}^{t}),f_{i}\neq f_{g}\\ x_{d}^{t+1}+K(\frac{x_{id}^{t}-{xw}_{d}^{t}}{|f_{i}-f_{w}|+e}),f_{i}=f_{g} \end{array}$$ (12)

where *xb*_*d*_ is the globally optimal location, *β* represents a random digit obeying standard normal distribution, *K*∈[−1,1] represents a random number. *e* is a minimal constant for avoiding the situation in which the denominator equals 0, *f*_*i*_ is the fitness value of the current sparrow, *f*_*g*_ is the global optimal, and *f*_*w*_ represents the worst fitness values.

All in all, the sparrow population iterates based on the Eqs of (10)–(12). Once the conditions are met, the process of position update of the sparrow population ends.

### 3.6 The framework of the proposed model

**Fig 2:**
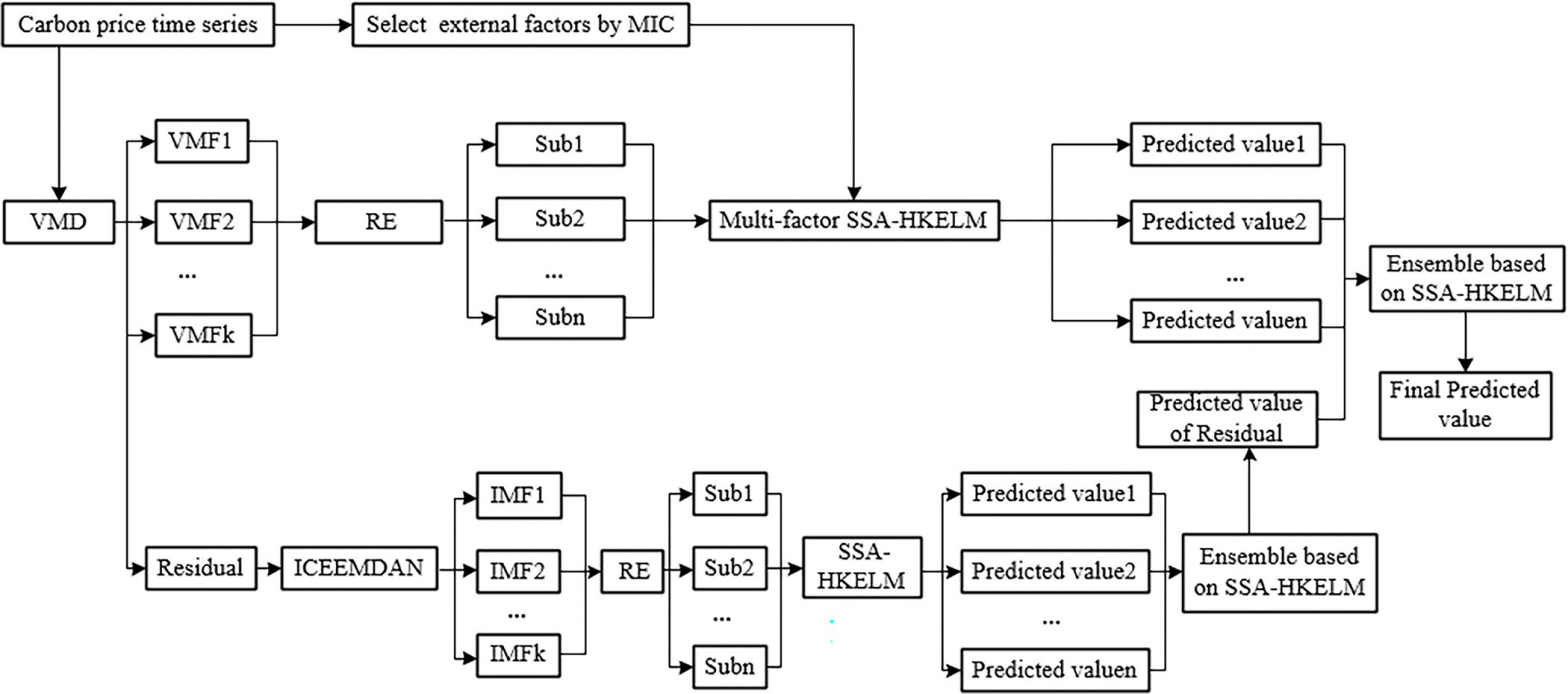
Framework of the developed model for predicting carbon price.

As China’s regional carbon price is affected by various internal and external factors, and has complex characteristics of fluctuations, such as nonstationarity and nonlinearity, this paper constructs a hybrid model including secondary decomposition strategy consisting of VMD-ICEEMDAN (SD) and ensemble learning, called the SD-RE-MIC-SSA-HKELM-Ensemble model, to predict the carbon price. The process of construction of the model is illustrated as Fig 2. According to Fig 2, the detailed steps are described as follows:

1. Decomposition of carbon price. Through VMD method, the original carbon price is decomposed into VMF components. The residual term is obtained by subtracting the sum of VMFs from the carbon price.
2. Reconstruction of VMFs. To achieve a balance between the cumulative error of prediction system and complexity of carbon price, the VMFs are reconstructed according to the RE. VMFs with low informational complexity are merged into a subseries and those with high informational complexity are regarded as a subsequence without merging.
3. Forecasting the reconstructed subsequences. To enhance the ability of each model to capture information, the main external factors, which impact China’s carbon price, and historical carbon prices are included in the SSA-HKELM model to forecast each reconstructed subsequence of VMFs. The main external factors influencing carbon price are selected by using the MIC and historical lags in each subsequence are analyzed by the PACF.
4. Forecasting the residual term. The residual term, which consists of time-series data containing complex information and irregular fluctuations, is forecasted by the three stages, namely, decomposition, reconstruction, and ensemble. Specifically, ICEEMDAN further decomposes the residual term into IMFs and reduce the complexity of the residual signal. RE is used to reconstruct the IMFs. The residual subsequences are predicted via the SSA-HKELM respectively. The predicted value of the residual term is then obtained via the SSA-HKELM-based ensemble learning, which means that the forecasted residual subsequences are taken as inputs.
5. Integrated prediction of carbon price. The final forecasts of carbon price are given by integrating the forecasting results of the above reconstructed subseries of VMFs and the residual term via the SSA-HKELM model, in which the weight distribution of the above forecasting results gets reasonable balance.

## 4 Empirical analysis

### 4.1 Sample selection and evaluation criteria

According to a 2021 market research report released by the Guangzhou Carbon Emission Exchange, it had traded rights to the emission of 277.558 million tons of carbon by the end of 2021, with a cumulative turnover of 5.276 billion yuan. This included 197.7 million tons of the GDEA spot, with the turnover of about 4.6 billion yuan, ranking first among pilot trading platforms in China [58]. The Guangzhou market is thus active, and its carbon price is highly representative of carbon markets across the country. We thus used the daily closing price of the GDEA spot in the Guangzhou carbon market as the sample data for the carbon price. The sample period ranged from January 3, 2017 to February 28, 2022. The sample size was 1184, the proportions of the training set and testing set in the paper are 80% and 20%. Specifically, the training dataset is the first 948 groups of data (2017/01/03-2021/03/08), and the last 236 groups (2021/03/09-2022/02/28) are test dataset. The data was collected from the Guangzhou Carbon Emission Exchange website(http://www.cnemission.cn, accessed on March 1, 2022). The MIC in this paper used the R4.1.3 software, and all other models were run using MATLAB2019b. The descriptive statistical result of the dataset is displayed as follows.

The value of standard deviation of Guangzhou’s carbon price in Table 1 is 12.69, which indicated that the data of the carbon price is relatively discrete and unstable. The range of carbon prices is approximately 84 yuan/ton of carbon dioxide, which indicated that the fluctuation range of the carbon price is relatively large and that the carbon price was widely dispersed. To sum up, the carbon market price in Guangzhou exhibited great volatility due to the impact of many factors.

**Table 1:** Statistical description of the dataset.

Market	Min	Max	Mean	Std	Kurtosis	Skewness
Guangzhou	11.05	95.26	24.54	12.69	6.89	1.62

Based on the analysis in section 3.4 and the availability of data, Table 2 details the variables representing external factors that influenced the carbon price.

**Table 2:** The multiple factors influencing carbon price.

Influencing factors	Index name	Factor symbol	unit	data sources
Domestic energy price	Qinhuangdao steam coal 5500	Coal1	Yuan / ton	wind
	Daqing crude oil	Oil1	Dollar/ barrel	wind
	The China LNG ex-works Price National Index	Gas1	Yuan / ton	wind
International energy price	Rotterdam coal futures price	Coal2	Dollar/ ton	wind
	Brent crude oil futures price	Oil2	Dollar/ barrel	wind
	UK natural gas futures price	Gas2	Pennies / Somme	wind
International carbon market	EUA futures carbon price	EUA	EUR / ton	wind
Macro economy	Shanghai securities composite index	SSEC	—	Investing.com
	Stoxx600	Stoxx	—	wind
weather conditions	Daily mean temperature in Guangzhou	TEM	Fahrenheit	NOAA
	Daily AQI in Guangzhou	AQI	—	wind

This paper selects three indexes to comprehensively test the effectiveness of the developed model. The evaluation indexes are displayed in Table 3, where *N* is the length of testing dataset, *y*_*i*_ is the actual carbon price, and ${\hat{y}}_{i}$ represents the forecasted data.

**Table 3:** The formulas of the evaluation indexes.

Index	RMSE	MAE	MAPE
**Formula**	$RMSE=\sqrt{\frac{1}{N}\sum_{i=1}^{N}{\left(y_{i}-{\overset{⌢}{y}}_{i}\right)}^{2}}$	$MAE=\frac{1}{N}\sum_{i=1}^{N}\left|y_{i}-{\overset{⌢}{y}}_{i}\right|$	$MAPE=\frac{1}{N}\sum_{i=1}^{N}\left|\frac{y_{i}-{\overset{⌢}{y}}_{i}}{y_{i}}\right|$

In addition, the Diebold–Mariano (DM) test was implemented for evaluating the prediction performance between the two models from a statistical point of view. In this study, the absolute prediction error serves as the loss function. The null hypothesis can be rejected when |DM|>Z_α/2_, α represents significance level. In addition, a detailed description of the DM test can be found in Diebold and Mariano (1995) [59].

### 4.2 Results of secondary decomposition and reconstruction

**Fig 3:**
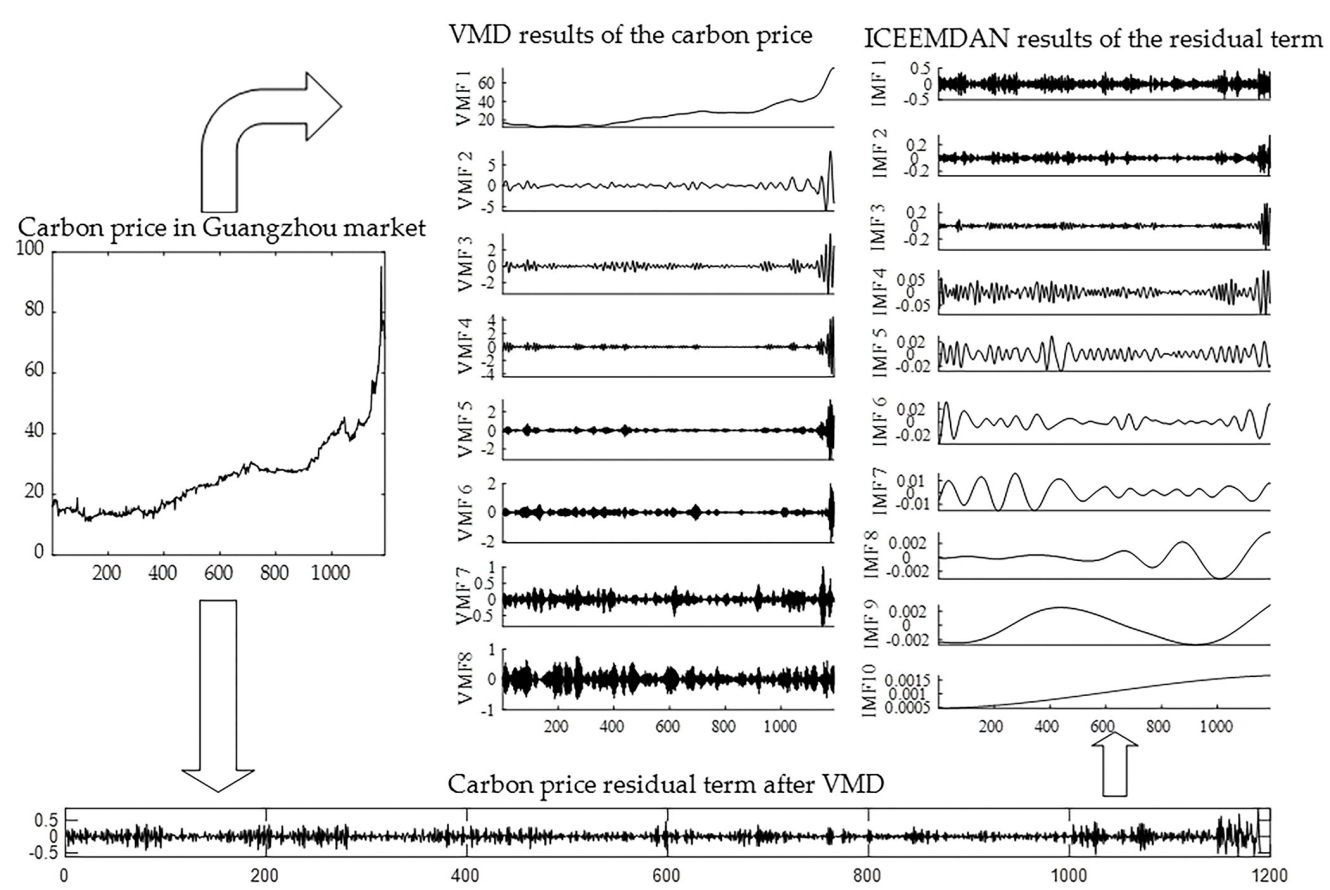
Results of secondary decomposition of the carbon price in the Guangzhou market.

The raw carbon price time series was essentially complex. The preprocessing of the carbon price could better extract the characteristics of the carbon price data. The VMD method was utilized to decompose the carbon price series of the Guangzhou market. K, which is the number of VMFs decomposed by VMD, should be reasonably preset. If K is quite small, the decomposition effect of VMD algorithm on carbon price is poor and the complexity of the carbon price struggles to be reduced. If there are too many decomposition layers, the frequencies of some modes become consistent, and even overlap, resulting in excessive decomposition [60]. The K is preset to 8 by referring to ICEEMDAN algorithm, which can automatically decompose the carbon price in the sample period into 8 modes. Namely, the number of VMF components generated by VMD is 8. The other parameters of VMD are set as: the default value of the penalty parameter α is used: α = 2000; the convergence criterion *ε* is set to 10^−6^. The results of VMD are displayed in Fig 3. Once the carbon price had been decomposed by VMD, there is a residual term that fluctuated violently and contained part of the information on carbon price. Therefore, ICEEMDAN was further utilized to extract the IMFs of the residual term. The main parameters of the ICEEMDAN are set as follows: the standard deviation of the noise, the numbers of realization, and the iteration number are set to 0.2, 500, 5000, respectively. The decomposition process is shown as follows.

**Fig 4:**
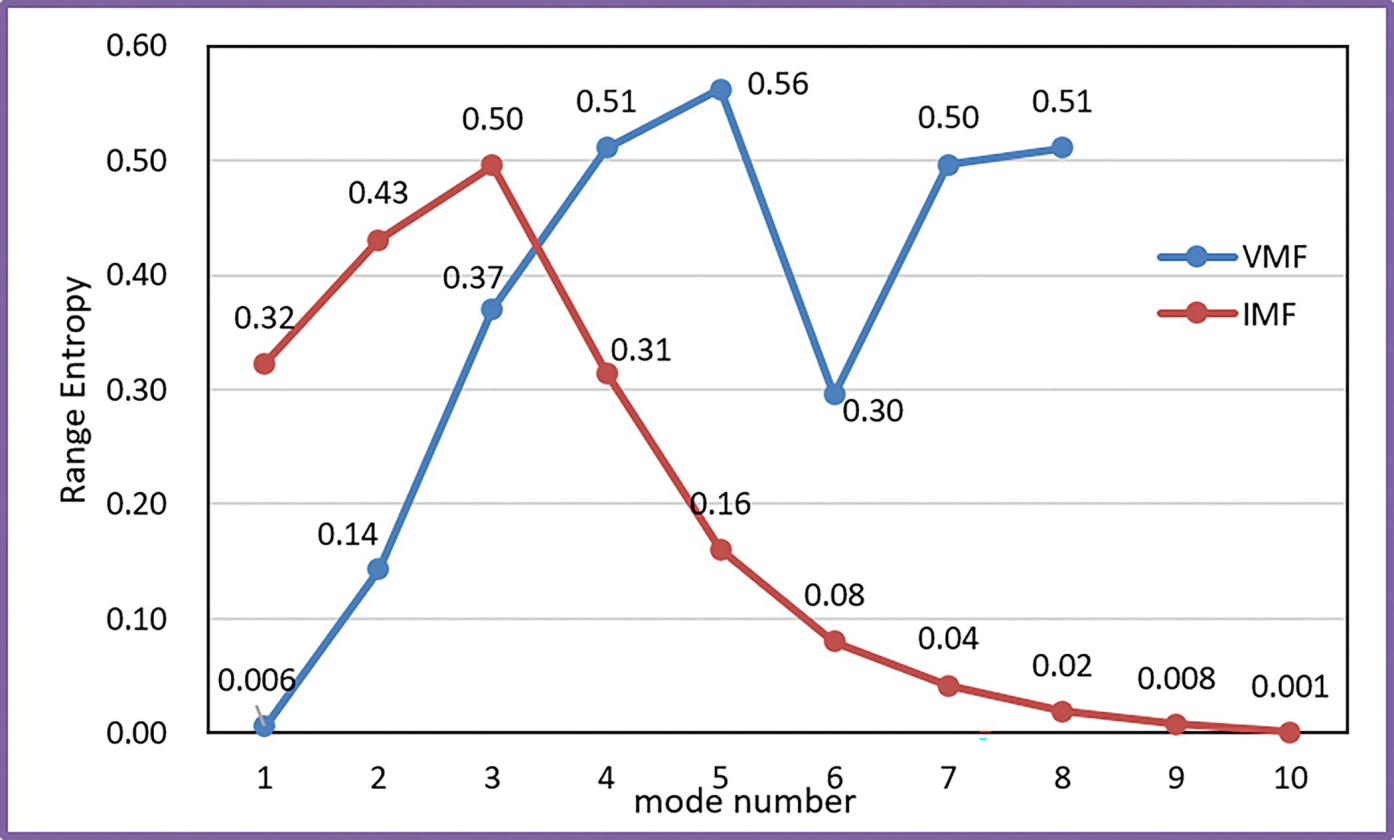
Analysis of the REs of VMFs and IMFs.

In Fig 3, the carbon prices in Guangzhou were decomposed into 18 subsequences, which represented the laws of different frequency levels of carbon prices. Namely, after the process of decomposition of carbon price by SD, a large number of subsequences were obtained. If these subsequences were directly predicted, the systematic error in the predicted carbon price would have been amplified. We thus reconstructed the subsequences to find a balance between the systematic error and the complexity of the data on carbon price. We reconstructed each VMF and IMF according to the RE. The results of RE are shown in Fig 4.

For the analysis of the RE of the VMFs and the IMFs: the RE values of the VMF1, VMF2, and IMF5-IMF10 were relatively low, which shows that they had long memory and were not complex. Therefore, this paper merged VMF1-VMF2 into a new subseries, and also merged IMF5-IMF10 into a new subseries. Obviously, RE values of other VMFs and IMFs were relatively high, indicating that their fluctuations were complicated, they were thus not merged but were predicted separately.

Eventually, 12 subseries were established after the reconstruction. The reconstruction of carbon price based on RE achieves the balance between prediction complexity and error accumulation. For convenience, the new merged subseries of VMF1-VMF2 is recorded as Sub1, the IMF1 to IMF4 are recorded as Sub8 to Sub11 in order, the merged subseries of IMF5-IMF10 is recorded as Sub12.

### 4.3 Selecting the input variables of the subseries of carbon price

#### 4.3.1 PACF results of the subseries of carbon price

**Fig 5:**
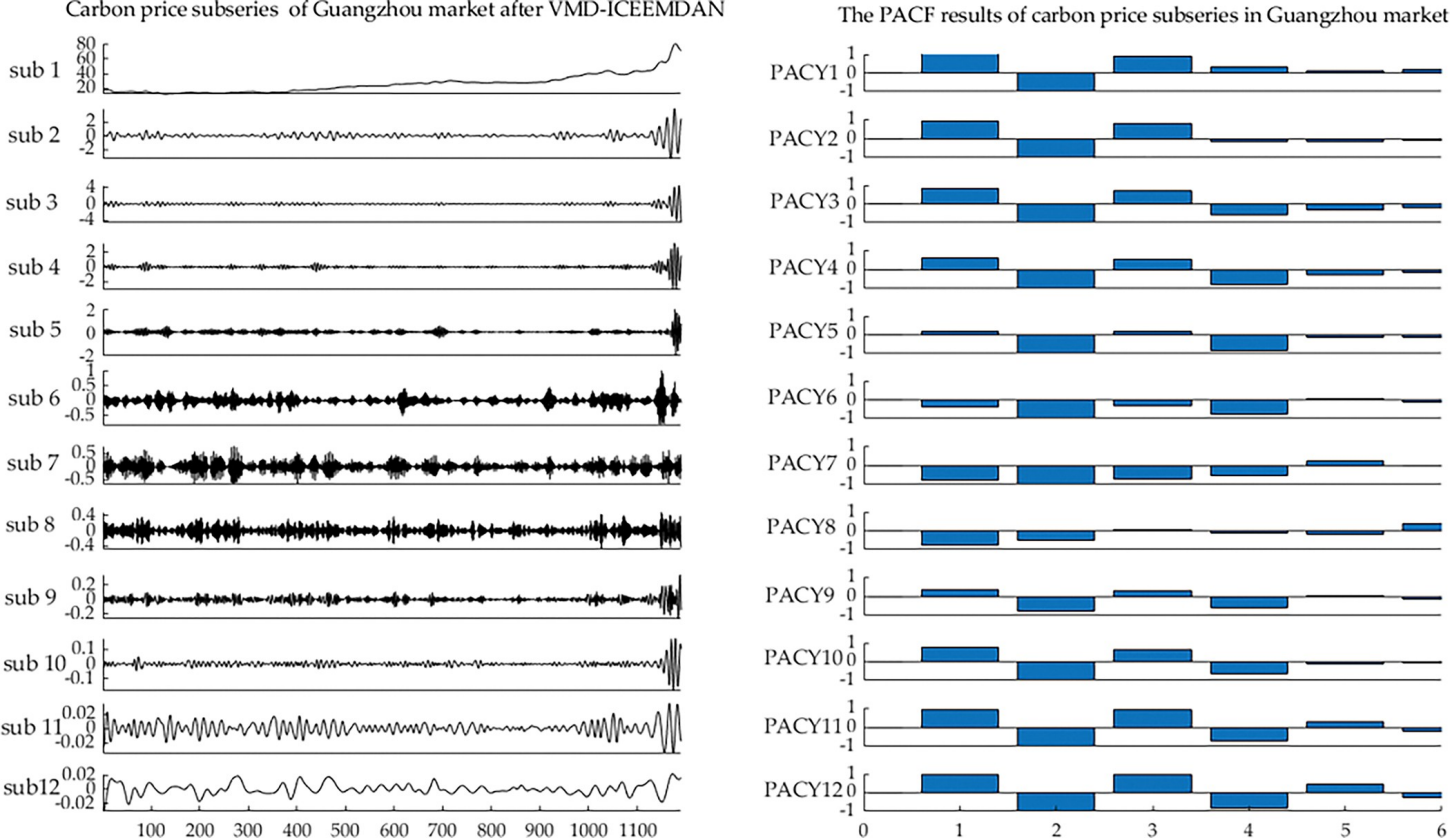
PACF results for the subseries of the carbon price.

The PACF was applied to describe the inherent correlation of the 12 subseries. The PACF results of the above reconstructed subseries from lags 1 to 6 are displayed in Fig 5, where the confidence level is 95%.

In Fig 5, each subsequence of carbon price is more regular, and is affected by its historical time series. Generally, the closer the historical data is, the greater the impact on the current subsequence of carbon price is. It makes clear that the historical carbon price includes useful information for predicting the current carbon price. Therefore, the lag period of each subsequence was part of the model input.

#### 4.3.2 Selection of external factors influencing carbon price

Because many external factors affect the carbon price, this paper used the MIC to select the main factors influencing it to lay the foundation for a subsequent model to predict carbon price. As the generalized elastic network (GEN) is an ideal method for measuring linear correlation, the results of the influence of various external factors on the carbon price using the GEN method are given for comparison. After deleting missing values, we normalized the data to [–1,1] to remove their dimensional influence, and then calculated the MIC between the influential factors in period t-1 and the carbon price in period t, as displayed in Table 4.

**Table 4:** Results of correlation between external factors and carbon price in Guangzhou.

Method	Coal1	Coal2	Oil1	Oil2	Gas1	Gas2	EUA	SSEC	Stoxx	TEM	AQI
MIC	0.63	0.92	0.54	0.55	0.44	0.70	0.79	0.59	0.68	0.45	0.21
GEN	0	0.06	0.57	-0.71	0	-0.03	0.69	0	0	-0.15	0.01

Based on the MIC, the carbon prices in the Guangzhou market shown in Table 4 were mostly affected by the price of Rotterdam coal and the EUA. First, the international price of coal exerted a significant impact on Guangzhou’s carbon price because the coal dominates China’s energy use. Moreover, Rotterdam coal is representative. Second, the price of EUA futures had the second greatest impact on the carbon price in Guangzhou. This is because the EUA price is representative of the international price of carbon. Compared with the mature EU carbon market, Guangzhou market lacks a systematic pricing mechanism and often refers to foreign carbon price. Therefore, the changes and fluctuations in the EUA price affected carbon price in Guangzhou. Finally, other factors had a certain influence on the carbon price that was, however, not significant. From the perspective of the GEN, the method had a good effect in terms of compressing irrelevant variables, and showed that Brent crude oil price and the EUA price had a higher linear impact on carbon price in Guangzhou. However, it could not capture the nonlinear relationship. Too many input variables in prediction model will reduce the performance of prediction. Therefore, we selected two influential factors, the price of Rotterdam coal and the EUA price, as part of the input variables to the model to predict the carbon price in Guangzhou.

The reconstructed subsequences of VMFs contained the major information on and the law of fluctuation in the carbon price. Therefore, predictions of these subsequences considered the main external factors influencing carbon price and the lag in each subseries as the input variables. This enhanced the capability of the prediction model to capture information on each subsequence of carbon price. However, the residual term after VMD fluctuated more and lacked definite rules. This was due to noise in data on the carbon market, and was predicted based only on the historical time series. Table 5 details the input variables to the prediction model for each subsequence.

**Table 5:** Input variables of prediction models of the subsequences.

Subseries	Input combination
Sub1	${{\mathrm{coal}2}_{t-1,}\mathrm{EUA}}_{t-1,}y_{t-1,}y_{t-2,}y_{t-3,}y_{t-4}$
Sub2	${{\mathrm{coal}2}_{t-1,}\mathrm{EUA}}_{t-1,}y_{t-1,}y_{t-2,}y_{t-3}$
Sub3-Sub7	${{\mathrm{coal}2}_{t-1,}\mathrm{EUA}}_{t-1,}y_{t-1,}y_{t-2,}y_{t-3,}y_{t-4}$
Sub8	$y_{t-1,}y_{t-2,}y_{t-6}$
Sub9-Sub11	$y_{t-1,}y_{t-2,}y_{t-3,}y_{t-4}$
Sub12	$y_{t-1,}y_{t-2,}y_{t-3,}y_{t-4,}y_{t-5}$

### 4.4 Forecasting process of carbon price

The reconstructed subsequences of VMFs and the residual term were predicted separately, and the final predicted carbon price was obtained through ensemble learning based on the SSA-HKELM. The map–min–max function was first used to normalize the dataset to [–1,1]. Then, the HKELM prediction model was employed to train and predict all the subsequences of carbon price. The HKELM model was trained according to the data of the training set of each subsequence. The optimal key parameters of the HKELM method for forecasting the subseries of carbon price were obtained through the SSA algorithm, which are illustrated as Table 6.

**Table 6:** Results of optimized parameters of the HKELM in the prediction stage of subseries.

Subseries	*L*	*C*	*a*	*b*	*d*
Sub1	0.005	782	507	927	1
Sub2	0.4	858	179	822	1
Sub3	0.5	999	355	0.003	1
Sub4	0.19	495	147	0.1	1
Sub5	0.04	405	382	504	1
Sub6	0.7	25	475	216	1
Sub7	0.5	61	144	297	1
Sub8	0.008	478	531	901	1
Sub9	0.91	838	622	342	1
Sub10	0.65	836	695	290	1
Sub11	0.05	920	679	409	1
Sub12	0.17	821	638	174	1

In the process of SSA optimizing the key parameters *L*, *C*, *a*, *b*, and *d*, the parameter settings for the SSA are as follows: the population size is set to 20, the maximum number of iterations is set to 20. The search range of *L* is [0, 1], the search range of *C*, *a*, and *b* is [0.001, 1000], and the search range of *d* is [1, 10]. It can be seen from Table 6 that the parameter *d* of each subsequence training is 1, which indicates that the complexity of the poly kernel function selected is low, and the value of *L* is greater than 0 and less than 1, which indicates that the weighted combination of poly and RBF kernel functions is applicable to each sub sequence fitting, not just one of them.

The data of the test set was then tested by using the SSA-HKELM to investigate the predictive effect of the model. Then comes the ensemble learning stage. Specifically, the SSA-HKELM was used as the ensemble tool to integrate the predicted values of residual subseries sub8–sub12 to get the results of prediction of the residual term. Finally, it was used again to integrate the predicted values of sub1–sub7 and the residual term to get the final prediction of the carbon price. The results of optimized parameters of the HKELM for ensemble learning of the residual term and the final carbon price are displayed in Table 7.

**Table 7:** Results of optimized parameter of the HKELM in the stage of ensemble learning.

Predicted sequence	*L*	*C*	*a*	*b*	*d*
Residual term	0.24	557	630	922	7
Final carbon price	0.14	421	915	792	10

It can be seen from Table 7 that in the integrated prediction stage of the residual term and the integrated prediction stage of the final carbon price, *L* is greater than 0, that is, the HKELM weighted by RBF kernel function and Poly kernel function is still used as the prediction model. In addition, the exponent *d* of poly kernel function is 7 and 10 respectively, indicating that the HKELM prediction model in the integration stage is relatively complex.

### 4.5 Comparative analysis of predictions of carbon price

**Fig 6:**
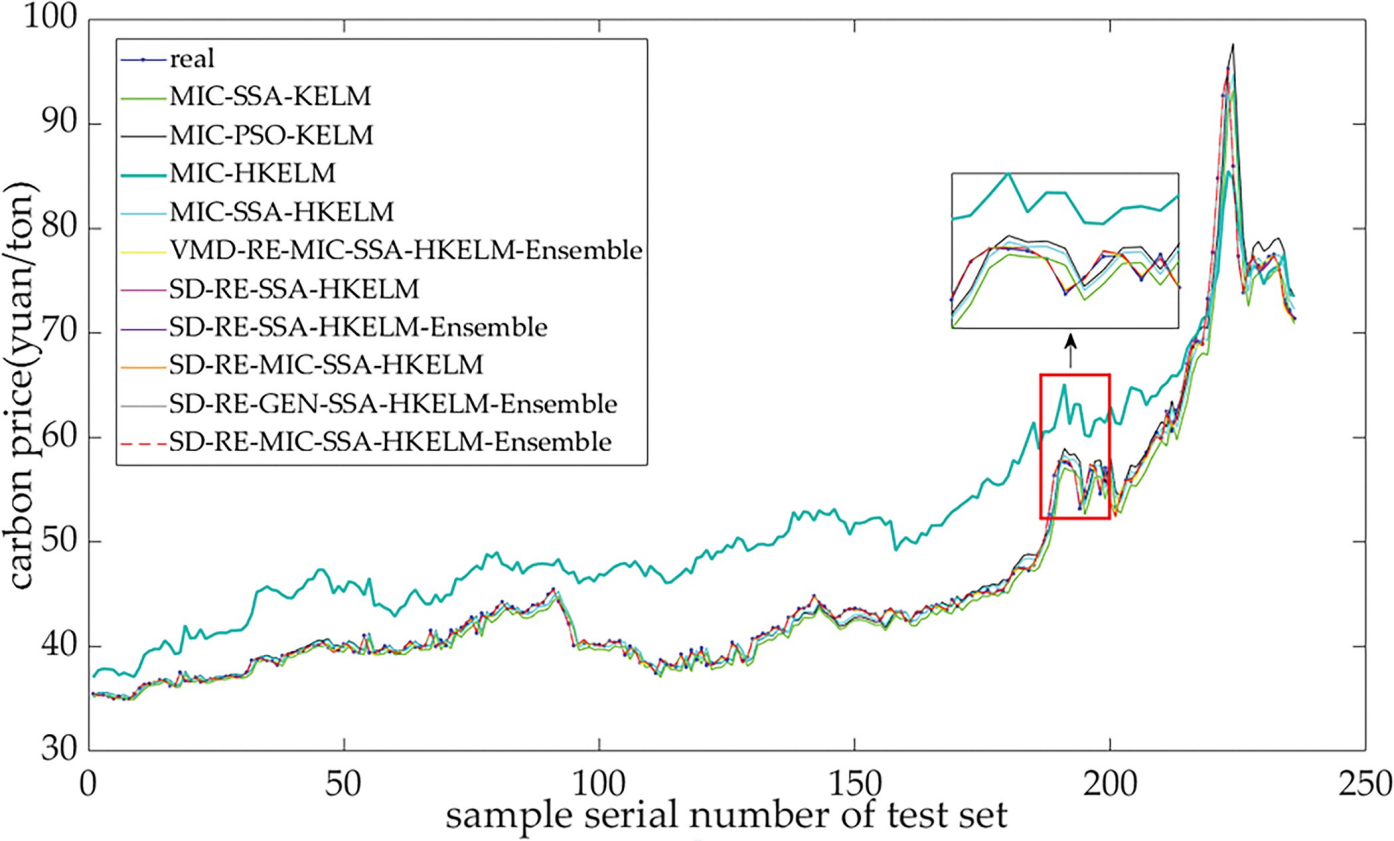
Forecasting results of different models for carbon price in Guangzhou market.

To investigate the performance of the proposed model for forecasting carbon price, this paper constructs eight benchmark models for comparison. More specifically, these benchmark models include single models, single decomposition-based model, and secondary decomposition-based models. Fig 6 and Table 8 reveal the comparison of the performances of these models about the forecasted carbon price in Guangzhou market.

**Table 8:** Comparison of performances of models for forecasting Guangzhou’s carbon price.

Models	RMSE	MAE	MAPE	DM
MIC-SSA-KELM	1.6572	1.0294	0.0199	10.949****
MIC-PSO-HKELM	1.5847	0.8774	0.0169	8.9562****
MIC-HKELM	6.7719	6.1590	0.1417	32.603****
MIC-SSA-HKELM	1.4598	0.8160	0.0157	8.9914 ****
VMD- RE-MIC-SSA-HKELM-Ensemble	0.2159	0.1497	0.0031	4.3140 ****
SD-RE-SSA-HKELM	0.1879	0.1331	0.0028	1.8577**
SD-RE-SSA-HKELM-Ensemble	0.1857	0.1312	0.0027	1.5927*
SD-RE-MIC-SSA-HKELM	0.1774	0.1251	0.0026	1.4447*
SD-RE-GEN-SSA-HKELM-Ensemble	0.1809	0.1293	0.0027	1.5307*
SD-RE-MIC-SSA-HKELM-Ensemble	0.1716	0.1218	0.0026	-

****, ** and * Indicates the 1%, 5% and 15% significance level respectively.

In Fig 6 and Table 8, the MIC-SSA-KELM model uses RBF kernel function. The MIC-HKELM uses the hybrid kernel function and does not optimize its parameters, where the parameters *L*, *a*, *b*, *d*, and *C* are set to 0.5, 1, 1, 1, and 1000, respectively. The SD means that a hybrid model including secondary decomposition-based strategy consisting of VMD-ICEEMDAN. The SD-RE-SSA-HKELM-Ensemble model does not consider external influential factors, the SD-RE-MIC-SSA-HKELM model does not carry out ensemble learning, and the SD-RE-GEN-SSA-HKELM-Ensemble model takes the EUA price and the price of Brent crude oil as the main influential factors and uses GEN rather than MIC for selecting the external factors.

Fig 6 shows that the forecasting results of the proposed model were closer to the actual price of carbon in Guangzhou. In Table 8, the RMSE, MAE, and MAPE of the proposed model were 0.1716, 0.1218, and 0.0026, respectively, and its error was not larger than those of the reference group models. This indicated that the predictive accuracy of this proposed model was higher than the other models considered. For the DM test, the values of the DM test were all larger than the Z_0.15/2_ = 1.44, indicating that the SD-RE-MIC-SSA-HKELM-Ensemble method outperformed other compared methods. The DM value of some models is small, possibly due to the slightly smaller difference between the prediction accuracy of the proposed model and the comparison model. However, it cannot deny the advantages of the proposed model compared to the comparative model.

The performance of predictions of the models is compared as follows: (1) The predictive accuracy of the proposed model was significantly better than that of SD -RE-SSA-HKELM-Ensemble, which was based only on the historical time series of carbon price. The reason is that the MIC algorithm can effectively select main external factors for the carbon price, which is critical for the success of the proposed model. This indicates that the introduction of the EUA price and the price of Rotterdam coal, to explain fluctuations in the carbon price, rendered predictions of the carbon price more accurate. (2) The model based on MIC-based variable selection was better than that based on GEN-based variable selection because China’s regional carbon price obeys a nonlinear series. The MIC is applicable to the variable selection of the factors influencing the nonlinear time series to screen out factors that exert the greater impact on China’s regional carbon price, while the GEN is based on a linear relationship, and struggles to adapt to the nonlinear characteristics of regional carbon price. (3) Predictions of the model based on the nonlinear integration of the SSA-HKELM were better than those of the direct linear superposition-based model SD-RE-MIC-SSA-HKELM. This is because the SSA-HKELM was used to integrate the residual subsequence of carbon price, and the finally predicted carbon price was obtained through an integrated prediction based on the SSA-HKELM. This could describe the impacts of different subsequences on predictions of the carbon price and obtain the best prediction. (4) The prediction of the proposed model was superior to those of the SD-RE-SSA-HKELM model proposed in the literature. This is because the proposed model predicted the reconstructed sequences of VMFs, considered external factors and the influence of the historical data of each subseries, and used SSA-HKELM as ensemble learning tool to get the results of prediction. (5) The predictions of the secondary decomposition-based models outperformed those of the single decomposition-based model because the residual term was too complex. The effect of using a single model to predict the residual term was poor. To reduce the prediction difficulty of the residual term, it was further decomposed and reconstructed. This provided a better representation of the law of change and frequency of the residual term for carbon price, and thus improved the predictive accuracy of the residual. (6) The combined model for forecasting the carbon price was superior to all single models. This is because any single model cannot comprehensively portray the complicated features of the carbon price. The combined model introduced decomposition technology to reduce the complexity of the carbon price. (7) Among single prediction models, the MIC-SSA-HKELM was superior. The possible reason was that the SSA algorithm can obtain the most effective key parameters for the HKELM model, thus avoiding the subjectivity of the artificial selection of parameters. It also demonstrated that the HKELM outperformed the KELM. Furthermore, the RMSE, MAE, and MAPE of the MIC-HKELM model were larger than other models. It indicated that the prediction effect of the model without optimization is very poor.

In summary, the proposed model to predict regional carbon price in China, which combines multi-factor HKELM, secondary decomposition and ensemble learning, not only helped analyze the factors influencing carbon price, but also significantly improved the accuracy of forecasting of the carbon price. This shows that this model is appropriate for predicting the regional carbon price in China.

### 4.6 Robustness

Testing the proposed model’s validity and applicability in only one regional carbon market is insufficient. Therefore, daily data from the Hubei carbon emission exchange was selected as a sample to fit and predict. The time range of the carbon price ranged from January 3, 2017 to February 28, 2022. The size of this dataset is 1,228 groups. The first 983 groups were taken as the training set and the other 245 as the testing set. As for external factor variables, AQI and temperature are the data of Wuhan, where Hubei’s carbon market is located. For other external variables, this paper selects the same data as the above Guangzhou market. The results of correlation between external factors and carbon price in Hubei are detailed in Table 9. According to the MIC, the carbon price in Hubei was mostly affected by the prices of Rotterdam coal and EUA. The GEN method showed that the prices of EUA and UK gas had a higher linear impact on carbon price in Hubei.

**Table 9:** Results of correlation between external factors and carbon price in Hubei.

Method	Coal1	Coal2	Oil1	Oil2	Gas1	Gas2	EUA	SSEC	Stoxx	TEM	AQI
MIC	0.45	0.57	0.36	0.39	0.36	0.49	0.85	0.41	0.38	0.27	0.14
GEN	-0.01	0.03	-0.02	0	0	-0.06	0.57	-0.01	-0.01	0.03	0

**Fig 7:**
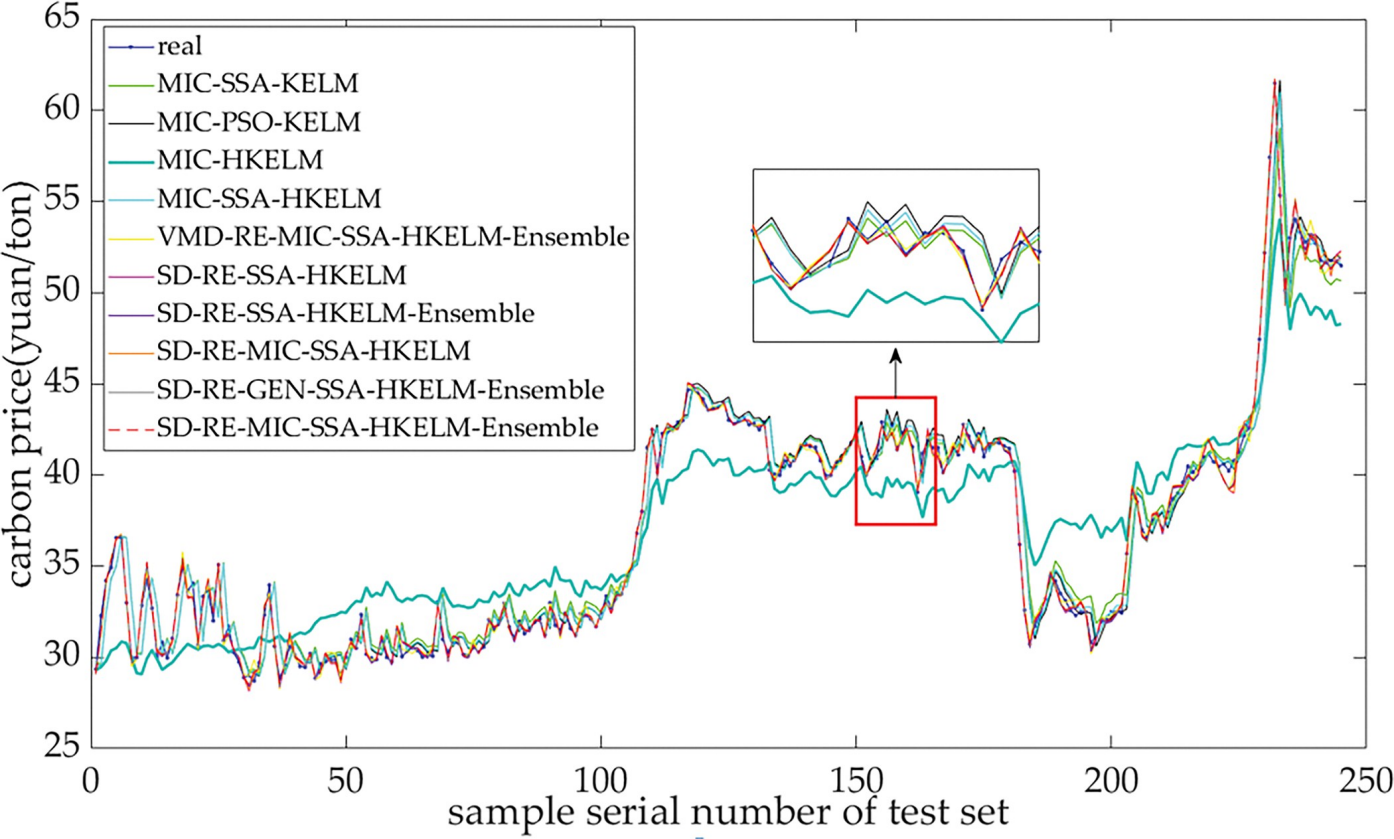
Forecasting results of different models for carbon price in Hubei market.

The results of prediction are displayed in Fig 7 and a comparison of the models is detailed in Table 10. There is no obvious change in the relative sizes of the values of these indexes including RMSE, MAE, MAPE, and DM test, indicating that the above models had good robustness.

**Table 10:** Comparison of performances of models for forecasting Hubei’s carbon price.

Models	RMSE	MAE	MAPE	DM
MIC-SSA-KELM	1.5037	1.0494	0.0289	11.35****
MIC-PSO-HKELM	1.4719	1.0100	0.0276	10.818****
MIC-HKELM	2.7964	2.3848	0.0657	22.711****
MIC-SSA-HKELM	1.4493	0.9849	0.0271	10.556****
VMD- RE-MIC-SSA-HKELM-Ensemble	0.3641	0.2750	0.0076	1.9527*
SD-RE-SSA-HKELM	0.3681	0.2743	0.0075	3.3453****
SD-RE-SSA-HKELM-Ensemble	0.3405	0.2616	0.0072	2.6427****
SD-RE-MIC-SSA-HKELM	0.3631	0.2694	0.0074	2.8928****
SD-RE-GEN-SSA-HKELM-Ensemble	0.3283	0.2506	0.0069	1.4663*
SD-RE-MIC-SSA-HKELM-Ensemble	0.3274	0.2492	0.0069	-

****, ** and * Indicates the 1%, 5% and 15% significance level respectively.

## 5 Conclusions

Predicting the regional carbon price in China is a crucial part of research on the carbon market. The current secondary decomposition-based prediction strategy, which obtains the final results of predictions of carbon price through equal weights-based linear superposition, ignores the impact of each subsequence on the results of predictions of carbon price, and the HKELM based only on historical time series of carbon price struggles to capture these complicated fluctuations in the carbon price in China. This paper constructed a combined model to predict carbon price based on the multi-factor HKELM, which combined secondary decomposition and ensemble learning, and verified its performance by testing it on carbon price in Guangzhou. The VMD and RE method were applied to decompose and reconstruct the carbon price into several subseries, and the SSA-HKELM model was employed to predict each subseries. The lags in each subseries of carbon price were selected by PACF and the external factors influencing carbon price were selected by using the MIC. The main external factors influencing carbon price and the historical time-series data of each subseries were taken as input variables to the prediction model. Given that the residual term after VMD consisted of time-series data with complex laws of fluctuation, ICEEMDAN and RE were used to decompose and reconstruct it, respectively. The prediction of the residual term was obtained through prediction and ensemble learning based on the SSA-HKELM. Finally, the final result of forecasting of the carbon price was obtained by integrating the results of predictions of the reconstructed subseries of VMFs and the residual term, with the SSA-HKELM as the ensemble learning method. Empirical comparative analysis showed that the Rotterdam coal price and EUA price are helpful in the prediction of China’s carbon price and the proposed model outperforms the models of the reference group in terms of prediction of the carbon price.

Compared with prevalent models for predicting China’s regional carbon price, the proposed model has certain advantages: (1) The multi-factor HKELM model considers the impact of external factors on carbon price, which enhances its explanatory power, makes it more economical, and improves the accuracy of prediction. (2) The MIC was introduced to identify the main external factors influencing the carbon price. It can reduce the complexity of the model used to forecast carbon price. (3) The final prediction of carbon price was obtained through SSA-HKELM-based ensemble learning, and the influence of different subsequences on the overall prediction of carbon price was considered.

Based on the above analysis results, this paper puts forward some policy suggestions. First of all, the evidence in this paper shows that the government should pay attention to the fluctuation of the international coal price in Rotterdam and develop more coal price risk management tools to reduce the risk contagion of coal price, because it has a greater impact on the regional carbon price in China. Secondly, in view of the high maturity of the EU carbon market and the relatively perfect price mechanism, policy makers also need to pay attention to the impact of EU carbon prices on the formation of China’s carbon market prices in the development process of China’s carbon market.

However, this study still has certain limitations. First, it was difficult to include the factors influencing major events in the proposed model. Appropriate methods should be used to quantify major events and expand the proposed method by combining it with the multi-factor prediction model in future work. Second, the empirical research only contained the regional carbon price in China. The combined model can be applied in the field of the EU carbon market price in future research.

## References

[1] Liu J.; Zhang Y. Has carbon emissions trading system promoted non-fossil energy development in China? Appl. Energy 2021, 302, 117613.

[2] Wen Y.; Hu P.; Li J.; Liu Q.; Shi L.; Ewing J.; Ma Z. Does China’s carbon emissions trading scheme really work? A case study of the Hubei pilot. J. Clean. Prod. 2020, 277, 124151.

[3] Lin B.; Huang C. Analysis of emission reduction effects of carbon trading: Market mechanism or government intervention? Sustain. Prod. Consump. 2022, 33, 28–37.

[4] Yun P.; Zhang C.; Wu Y.; Yang Y. Forecasting carbon dioxide price using a time-varying high-order moment hybrid model of NAGARCHSK and gated recurrent unit network. Int. J. Environ. Res. Public Health 2022, 19,899. doi: 10.3390/ijerph19020899 35055721 PMC8775960

[5] Pradhan B. K.; Ghosh J.; Yao Y.; Liang Q. Carbon pricing and terms of trade effects for China and India: A general equilibrium analysis. Econ. Model. 2017, 63, 60–74.

[6] Wu Q.; Wang Y. How does carbon emission price stimulate enterprises’ total factor productivity? Insights from China’s emission trading scheme pilots. Energy Econ. 2022, 109, 105990.

[7] Zhao L.; Miao J.; Qu S.; Chen X. A multi-factor integrated model for carbon price forecasting: Market interaction promoting carbon emission reduction. Sci. Total Environ. 2021,796,149110. doi: 10.1016/j.scitotenv.2021.149110 34328877

[8] Yang H.; Yang X.; Li G. Forecasting carbon price in China using a novel hybrid model based on secondary decomposition, multi-complexity and error correction. J. Clean. Prod. 2023, 401,136701.

[9] Ren C.; Lo A.Y. Emission trading and carbon market performance in Shenzhen, China. Appl. Energy 2017, 193, 414–425.

[10] Huang Y.; Hu J.; Liu H.; Liu S. Research on price forecasting method of China’s carbon trading market based on PSO-RBF algorithm. Syst. Sci. Control. Eng. 2019, 7, 40–47.

[11] Wen F.; Zhao H.; Zhao L.; Yin H. What drive carbon price dynamics in China? Int. Rev. Financ. Anal. 2022, 79, 101999.

[12] Li W, Lu C. The research on setting a unified interval of carbon price benchmark in the national carbon trading market of China. Appl. Energy 2015, 155, 728–739.

[13] Sun W.; Xu C. Carbon price prediction based on modified wavelet least square support vector machine. Sci. Total Environ. 2021, 754,142052. doi: 10.1016/j.scitotenv.2020.142052 32916491

[14] Wang J.; Sun X.; Cheng Q.; Cui Q. An innovative random forest-based nonlinear ensemble paradigm of improved feature extraction and deep learning for carbon price forecasting. Sci. Total Environ. 2021, 762,143099. doi: 10.1016/j.scitotenv.2020.143099 33127140

[15] Liang Y.; Lin Y.; Lu Q. Forecasting gold price using a novel hybrid model with ICEEMDAN and LSTM-CNN-CBAM. Expert Syst. Appl. 2022, 206,117847.

[16] Zhou J.; Chen D. Carbon Price forecasting based on improved CEEMDAN and extreme learning machine optimized by sparrow search algorithm. Sustainability 2021, 13, 4896.

[17] Niu X.; Wang J.; Zhang L. Carbon price forecasting system based on error correction and divide-conquer strategies. Appl. Soft. Comput. 2022, 118,107935.

[18] Wang J.; Cui Q.; He M. Hybrid intelligent framework for carbon price prediction using improved variational mode decomposition and optimal extreme learning machine. Chaos Solitons Fractals 2022, 156,111783.

[19] Sun J.; Zhao P.; Sun S. A new secondary decomposition-reconstruction-ensemble approach for crude oil price forecasting. Resources Policy 2022, 77,102762.

[20] Zhou F.; Huang Z.; Zhang C. Carbon price forecasting based on CEEMDAN and LSTM. Appl. Energy 2022,311,118601.

[21] Li G.; Ning Z.; Yang H.; Gao L. A new carbon price prediction model. Energy 2022, 239, 122324.

[22] Cheng Y.; Hu B. Forecasting Regional Carbon Prices in China Based on Secondary Decomposition and a Hybrid Kernel-Based Extreme Learning Machine. Energies 2022, 15, 3562.

[23] Lei H.; Xue M.; Liu H. Probability distribution forecasting of carbon allowance prices: A hybrid model considering multiple influencing factors. Energy Econ. 2022,113,106189.

[24] Lutz B. J.; Pigorsch U.; Rotfuß W. Nonlinearity in cap-and-trade systems: The EUA price and its fundamentals. Energy Econ. 2013, 40, 222–232.

[25] Lovcha Y.; Laborda A.; Sikora I. The determinants of CO2 prices in the EU emission trading system. Appl. Energy 2022, 305,117903.

[26] Guo L.; Feng C.; Yang J. Can energy predict the regional prices of carbon emission allowances in China? Int. Rev. Financ. Anal. 2022, 82, 102210.

[27] Zeng S.; Nan X.; Liu C.; Chen J. The response of the Beijing carbon emissions allowance price (BJC) to macroeconomic and energy price indices. Energy Policy 2017, 106, 111–121.

[28] Sun W.; Zhang J. A novel carbon price prediction model based on optimized least square support vector machine combining characteristic-scale decomposition and phase space reconstruction. Energy 2022, 253, 124167.

[29] Lin B.; Xu B. A non-parametric analysis of the driving factors of China’s carbon prices. Energy Econ. 2021,104,105684.

[30] Zhou J.; Wang S. A carbon price prediction model based on the secondary decomposition algorithm and influencing factors. Energies 2021, 14, 1328.

[31] Hao Y.; Tian C. A hybrid framework for carbon trading price forecasting: The role of multiple influence factor. J. Clean. Prod. 2020, 262, 120378.

[32] Han M.; Ding L.; Zhao X.; Kang W. Forecasting carbon prices in the Shenzhen market, China: The role of mixed-frequency factors. Energy 2019, 171, 69–76.

[33] Xie Q.; Hao J.; Li J.; Zheng X. Carbon price prediction considering climate change: A text-based framework. Econ. Anal. Policy 2022, 74, 382–401.

[34] Sun G.; Li J.; Dai J.; Song Z.; Lang F. Feature selection for IoT based on maximal information coefficient. Futur. Gener. Comp. Syst. 2018, 89, 606–616.

[35] Dragomiretskiy K.; Zosso D. Variational Mode Decomposition[J]. IEEE Trans. Signal Process. 2014, 62, 531–544.

[36] Liu S.; Zhao R.; Yu K.; Zheng B.; Liao B. Output-only modal identification based on the variational mode decomposition (VMD) framework. J. Sound and Vibr. 2022, 522,116668.

[37] Zhao X.; Wu P.; Yin X. A quadratic penalty item optimal variational mode decomposition method based on single-objective salp swarm algorithm. Mech. Syst. Signal Proc. 2020, 138, 106567.

[38] Emeksiz C.; Tan M. Wind speed estimation using novelty hybrid adaptive estimation model based on decomposition and deep learning methods (ICEEMDAN-CNN). Energy 2022, 249,123785.

[39] Omidvarnia A.; Mesbah M.; Pedersen M.; Jackson G. Range entropy: A bridge between signal complexity and self-similarity. Entropy 2018, 20, 1–22. doi: 10.3390/e20120962 33266686 PMC7512560

[40] Chang K.; Ge F.; Zhang C.; Wang W. The dynamic linkage effect between energy and emissions allowances price for regional emissions trading scheme pilots in China. Renew. Sustain. Energy Rev. 2018, 98, 415–425.

[41] Tao J.; Ferreira J.; González S.E. New insights into decoupling economic growth, technological progress and carbon dioxide emissions: Evidence from 40 countries. Technol. Forecast. Soc. Chang. 2022, 174, 121250.

[42] Lv J.; Fan X.; Wu H. Sensitivity Analysis of Factors Influencing Carbon Prices in China. Soft Science 2021, 35, 123–130.

[43] Yang B.; Liu C.; Gou Z.; Man J.; Su Y. How Will Policies of China’s CO2 ETS Affect its Carbon Price: Evidence from Chinese Pilot Regions. Sustainability 2018, 10, 605.

[44] Reshef D. N.; Reshef Y. A.; Finucane H.K.; Grossman S.R.; McVean G.; Turnbaugh P.J.; et al. Detecting Novel Associations in Large Data Sets. Science 2011, 334, 1518–1524. doi: 10.1126/science.1205438 22174245 PMC3325791

[45] Wu J.; Li N.; Zhao Y.; Wang J. Usage of correlation analysis and hypothesis test in optimizing the gated recurrent unit network for wind speed forecasting. Energy 2022, 242,122960.

[46] He Y.; Li J.; Ruan S.; Zhao S. A Hybrid Model for Financial Time Series Forecasting-Integration of EWT, ARIMA with The Improved ABC Optimized ELM. IEEE Access 2020, 8, 84501–84518.

[47] Liu D.; Li M.; Wang K.; Fu Q.; Zhang L.; Li M.; Li X.; Li T.; Cui S. Evaluation and analysis of irrigation water use efficiency based on an extreme learning machine model optimized by the spider monkey optimization algorithm. J. Clean. Prod. 2022, 330,129935.

[48] Huang G.; Zhou H.; Ding X.; Zhang R. Extreme learning machine for regression and multiclass classification. IEEE Trans. Syst. Man Cybern. Part B-Cybern. 2012, 42, 513–529. doi: 10.1109/TSMCB.2011.2168604 21984515

[49] Fu W.; Wang K.; Tan W.; Zhang K. A composite framework coupling multiple feature selection, compound prediction models and novel hybrid swarm optimizer-based synchronization optimization strategy for multi-step ahead short-term wind speed forecasting. Energy Conv. Manag. 2020, 205,112461.

[50] Rayi V.K.; Mishra S.P.; Naik J.; Dash P.K. Adaptive VMD based optimized deep learning mixed kernel ELM autoencoder for single and multistep wind power forecasting. Energy 2022, 244, 122585.

[51] Xie Z.; Wu Z. Maximum power point tracking algorithm of PV system based on irradiance estimation and multi-Kernel extreme learning machine. Sustain. Energy Technol. Assess. 2021, 44,101090.

[52] Wang Z.; Chen H.; Wang M.; Zhang X.; Dou Y. Solid particle erosion prediction in elbows based on machine learning and swarm intelligence algorithm. J. Pet. Sci. Eng. 2022, 218,111042.

[53] Salb M.; Zivkovic M.; Bacanin N.; Chhabra A.; Suresh M. Support Vector Machine Performance Improvements for Cryptocurrency Value Forecasting by Enhanced Sine Cosine Algorithm. In: Bansal J.C., Engelbrecht A., Shukla P.K. (eds) Computer Vision and Robotics. Algorithms for Intelligent Systems. Springer, Singapore. 2022, pp 527–536.

[54] Bacanin N.; Stoean C.; Zivkovic M.; Rakic M.; Strulak-Wójcikiewicz R.; Stoean R. On the Benefits of Using Metaheuristics in the Hyperparameter Tuning of Deep Learning Models for Energy Load Forecasting. Energies 2023, 16, 1434.

[55] Jovanovic L.; Jovanovic D.; Bacanin N.; Jovancai Stakic A.; Antonijevic M.; Magd H.; et al. Multi-Step Crude Oil Price Prediction Based on LSTM Approach Tuned by Salp Swarm Algorithm with Disputation Operator. Sustainability 2022, 14, 14616.

[56] Xue J K, Shen B. A novel swarm intelligence optimization approach: Sparrow search algorithm. Sys. Sci. Control. Eng. 2020, 8, 22–34.

[57] Li J.; Lei Y.; Yang S. Mid-long term load forecasting model based on support vector machine optimized by improved sparrow search algorithm. Energy Reports 2022, 8, 491–497.

[58] Wu Q. Price and scale effects of China’s carbon emission trading system pilots on emission reduction. J. Environ. Manage. 2022, 314, 115054. doi: 10.1016/j.jenvman.2022.115054 35430515

[59] Diebold F.X.; Mariano R.S. Comparing Predictive Accuracy. J. Bus. Econ. Stat. 1995,13, 253–263.

[60] Li H.; Wu X.; Liu T.; Li S.; Zhang B.; Zhou G.; Huang T. Composite fault diagnosis for rolling bearing based on parameter-optimized VMD. Measurement 2022, 201,111637.

